# An occurence records database of Irregular Echinoids (Echinodermata: Echinoidea) in Mexico

**DOI:** 10.3897/BDJ.4.e7729

**Published:** 2016-07-07

**Authors:** Alejandra Martínez-Melo, Francisco Alonso Solís-Marín, Blanca Estela Buitrón-Sánchez, Alfredo Laguarda-Figueras

**Affiliations:** ‡ Insituto de Geología, Universidad Nacional Autónoma de México, Mexico, Mexico; § Instituto de Ciencias del Mar y Limnología, Universidad Nacional Autónoma de México, Mexico, Mexico

**Keywords:** Gulf of California, Panamic Region, Bank of Campeche, Echinoneoida, Cassiduloida, Echinolampadoida, Clypeasteroida, Holasteroida, Spatangoida, Colección Nacional de Equinodermos "Ma. Elena Caso Muñoz".

## Abstract

**Background:**

Research on echinoderms in Mexico began in the late nineteenth century. We present a dataset that includes the taxonomic and geographic information of irregular echinoids from Mexico, housed in four collections: 1) Colección Nacional de Equinodermos “Ma. Elena Caso Muñoz” from the Instituto de Ciencias del Mar y Limnología (ICML), Universidad Nacional Autónoma de México (UNAM); 2) Invertebrate Zoology Collection, Smithsonian Museum of Natural History, Washington, D.C., United States of America (USA); 3) Invertebrate Collection, Museum of Comparative Zoology, University of Harvard, Boston, Massachusetts, USA and 4) Invertebrate Zoology, Peabody Museum, Yale University, New Haven, Connecticut, USA.

**New information:**

A total of six orders, 17 families, 35 genera and 68 species are reported, 37 distributed in the Pacific coast and 31 in the Atlantic coast, none of them was found in both coasts. The most diverse region is the Gulf of California (S=32); the most diverse order is Spatangoida with 31 species reported in mexican waters.

## Introduction

Research on echinoderms in Mexico started on 1838 with the report of *Mellita hexapora* in Veracruz by L. E. Agassiz and G. Valentin. During XIX century, the Challenger and Albatross expeditions collected specimens in mexican territory (Durán-González et al. 2005).

Caso published a series of monographs (Caso 1978, Caso 1980, Caso 1983) of echinoid species of the Pacific coast of Mexico, including diagnosis, synonyms, description, measurements, distribution, reports in Mexico and catalogue number for each species, as well as taxonomic keys for the order, family genus and species level. Bravo-Tzompantzi et al. 1999 reported the echinoids from Puerto Morelos, Quintana Roo, known as a diverse area for echinoderms. They present taxonomic keys, systematics, illustrations, as well as geographic and bathymetric data. Bravo-Tzompantzi et al. 2000 enlisted fossil and extant echinoid species from the Gulf of Mexico and the Mexican Caribbean Coast. This work described the problems around the economic importance and the legal aspects of using the natural resources of the Atlantic coast of Mexico.

Solís-Marín et al. 2013 reported 58 species of irregular echinoids in Mexico, analyzing the fauna in different marine regions, where the Gulf of Mexico was the most diverse region of echinoids in Mexico. A revision of echinoids form the Gulf of Mexico (Laguarda-Figueras et al. 2005a) reported species collected in different oceanographic expeditions, including geographic coordinates of the reports; that revision includes taxonomic keys for the echinoids from the Gulf of Mexico as well as photographs, synonyms, diagnosis, descriptions and distribution for each species. Similar works have been published for echinoderm species reported for different marine regions in Mexico: 51 species of echinoids in Gulf of Mexico (Durán-González et al. 2005), 36 species of echinoids in the Mexican Caribbean (Laguarda-Figueras et al. 2005b) and 40 species of echinoids in the Gulf of California (Solís-Marín et al. 2005).

In this work we present a dataset that includes the taxonomic and geographic information of irregular echinoids from Mexico; this data was submitted in a parsimony analysis endemicity (Martínez-Melo et al. 2014) resulting in four biogeographic regions in the Atlantic coast and five in the Pacific coast, suggesting that the oceanic currents and sediments are the environmental factors that determine the distribution of irregular echinoids in the Mexican Atlantic coast; on the other hand, oceanic currents and depth are the environmental factors that determine the distribution of irregular echinoids in the Mexican Pacific coast.

Mexico host an important diversity of echinoderms. 643 species have been reported in mexican territory, aboout 10% of the species of echinoderms reported in the world (Solís-Marín et al. 2013). Recognizing the mexican species has been possible due to taxonomic inventories of the phylum in different coastal habitats, represeting valuable information (Solís-Marín and Laguarda-Figueras 2012).

## Sampling methods

### Study extent

See spatial coverage and geographic coverage descriptions.

### Sampling description

This study includes irregular echinoids collections. Records of irregular echinoids were recovered from four biological collections:

1. National Echinoderms Collection “Ma. Elena Caso Muñoz”, Institute of Marine Sciences and Limnology (ICML), National Autonomous University of Mexico (UNAM).
2. Invertebrate Zoology Collection, Smithsonian Museum of Natural History, Washington, D.C., United States of America (USA).
3. Invertebrate Collection, Museum of Comparative Zoology, University of Harvard, Boston, Massachusetts, USA.
4. Invertebrate Zoology, Peabody Museum, Yale University, New Haven, Connecticut, USA.

### Quality control

We redetermined specimens at species level, and the species were classified under the criteria of Kroh and Smith 2010; sbspecies were included at species level. Names were verified against the Worls Register of Marine Species (WoRMS). Collection data were downloaded from the databases from 4 biologic collections (1.ICML-UNAM, 2.USNM, 3.MCZ, 4.YPM). and copied from specimens labels. Geographic data were corroborated with electronic maps by Ocean Biogeographic Information System (OBIS).

## Geographic coverage

### Description

**Figure 1.**
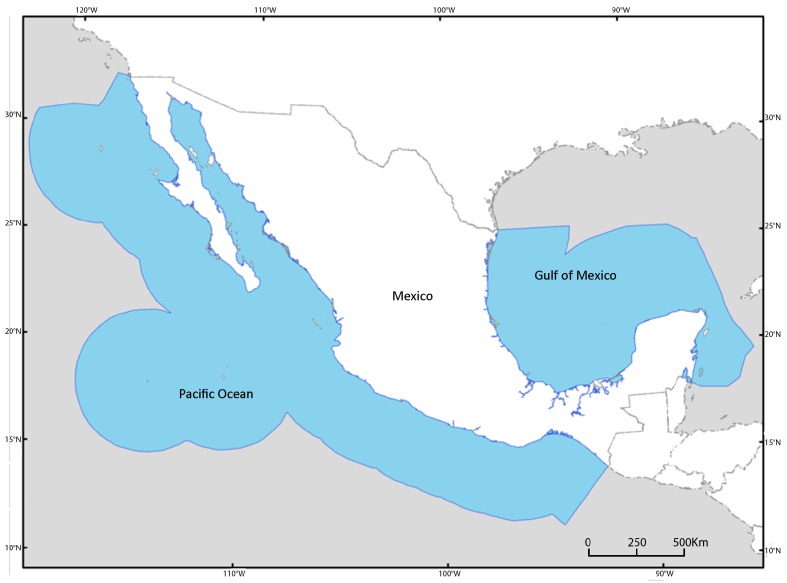
Economic Exclusive Zone of Mexico

This study covers the Economic Exclusive Zone of Mexico, including both coastlines Fig. 1

## Taxonomic coverage

### Description

This database concerns all irregular echinoid (Echinodermata: Echinoidea: Irregularia) species inhabiting Mexico. Species are listed in Table 1, including the scientific collections where material is hosted and the marine provinces (according to Aguayo and Trápaga 1996) where the species inhabits.

### Taxa included

**Table:** 

Rank	Scientific Name	
kingdom	Animalia	
phylum	Echinodermata	
class	Echinoidea	
subclass	Irregularia	

## Temporal coverage

**Living time period:** 1838 - 2014.

### Notes

This study includes reports of irregular echinoids collected in Mexico from different expeditions since 1838 to date.

## Collection data

### Collection name

Colección Nacional de Equinodermos "María Elena Caso Muñoz"

### Collection identifier

ICML-UNAM

### Specimen preservation method

Dry and ethanol alcohol.

## Usage rights

### Use license

Оpen Data Commons Open Database License (ODbL)

## Data resources

### Data package title

Occurence of Irregular Echinoids in mexican waters

### Number of data sets

1

### Data set 1.

#### Data set name

Irregularia

#### Data format

Darwin Core

#### Number of columns

37

#### Description

Suppl. material 1

**Data set 1.** 

Column label	Column description
dc:type	The nature or genre of the resource For Darwin Core, recommended best practice is to use the name of the class that defines the root of the record.
dc:language	A language of the resource Recommended best practice is to use a controlled vocabulary such as RFC 4646 [RFC4646].
dc:rightsHolder	A person or organization owning or managing rights over the resource
dc:accessRights	Information about who can access the resource or an indication of its security status Access Rights may include information regarding access or restrictions based on privacy, security, or other policies.
basisOfRecord	The specific nature of the data record - a subtype of the dcterms:type Recommended best practice is to use a controlled vocabulary such as the Darwin Core Type Vocabulary (http://rs.tdwg.org/dwc/terms/#basisOfRecord).
individualID	An identifier for an individual or named group of individual organisms represented in the Occurrence. Meant to accommodate resampling of the same individual or group for monitoring purposes. May be a global unique identifier or an identifier specific to a data set.
individualCount	The number of individuals represented present at the time of the Occurrence.
disposition	The current state of a specimen with respect to the collection identified in collectionCode or collectionID Recommended best practice is to use a controlled vocabulary.
Continent	The name of the continent in which the Location occurs Recommended best practice is to use a controlled vocabulary such as the Getty Thesaurus of Geographic Names or the ISO 3166 Continent code.
waterBody	The name of the water body in which the Location occurs Recommended best practice is to use a controlled vocabulary such as the Getty Thesaurus of Geographic Names.
iIslandGroup	The name of the island group in which the Location occurs Recommended best practice is to use a controlled vocabulary such as the Getty Thesaurus of Geographic Names.
island	The name of the island on or near which the Location occurs Recommended best practice is to use a controlled vocabulary such as the Getty Thesaurus of Geographic Names.
country	The name of the country or major administrative unit in which the Location occurs Recommended best practice is to use a controlled vocabulary such as the Getty Thesaurus of Geographic Names.
countryCode	The standard code for the country in which the Location occurs Recommended best practice is to use ISO 3166-1-alpha-2 country codes.
stateProvince	The name of the next smaller administrative region than country (state, province, canton, department, region, etc) in which the Location occurs.
municipality	The full, unabbreviated name of the next smaller administrative region than county (city, municipality, etc) in which the Location occurs Do not use this term for a nearby named place that does not contain the actual location.
locality	The specific description of the place Less specific geographic information can be provided in other geographic terms (higherGeography, continent, country, stateProvince, county, municipality, waterBody, island, islandGroup) This term may contain information modified from the original to correct perceived errors or standardize the description.
verbatimDepth	The original description of the depth below the local surface
verbatimLatitude	The verbatim original latitude of the Location The coordinate ellipsoid, geodeticDatum, or full Spatial Reference System (SRS) for these coordinates should be stored in verbatimSRS and the coordinate system should be stored in verbatimCoordinateSystem
verbatimLongitude	The verbatim original longitude of the Location The coordinate ellipsoid, geodeticDatum, or full Spatial Reference System (SRS) for these coordinates should be stored in verbatimSRS and the coordinate system should be stored in verbatimCoordinateSystem
verbatimCoordinateSystem	The spatial coordinate system for the verbatimLatitude and verbatimLongitude or the verbatimCoordinates of the Location Recommended best practice is to use a controlled vocabulary
verbatimSRS	The ellipsoid, geodetic datum, or spatial reference system (SRS) upon which coordinates given in verbatimLatitude and verbatimLongitude, or verbatimCoordinates are based Recommended best practice is use the EPSG code as a controlled vocabulary to provide an SRS, if known Otherwise use a controlled vocabulary for the name or code of the geodetic datum, if known Otherwise use a controlled vocabulary for the name or code of the ellipsoid, if known If none of these is known, use the value "unknown".
scientificName	The full scientific name, with authorship and date information if known When forming part of an Identification, this should be the name in lowest level taxonomic rank that can be determined This term should not contain identification qualifications, which should instead be supplied in the IdentificationQualifier term.
acceptedNameUsage	The full name, with authorship and date information if known, of the currently valid (zoological) or accepted (botanical) taxon.
parentNameUsage	The full name, with authorship and date information if known, of the direct, most proximate higher-rank parent taxon (in a classification) of the most specific element of the scientificName.
kingdom	The full scientific name of the kingdom in which the taxon is classified
phylum	The full scientific name of the phylum or division in which the taxon is classified.
class	The full scientific name of the class in which the taxon is classified.
order	The full scientific name of the order in which the taxon is classified.
family	The full scientific name of the family in which the taxon is classified.
genus	The full scientific name of the genus in which the taxon is classified.
specificEpithet	The name of the first or species epithet of the scientificName.
infraSpecificEpithet	The name of the lowest or terminal infraspecific epithet of the scientificName, excluding any rank designation.
taxonRank	The taxonomic rank of the most specific name in the scientificName Recommended best practice is to use a controlled vocabulary.
scientificNameAuthorship	The authorship information for the scientificName formatted according to the conventions of the applicable nomenclaturalCode.
nomenclaturalCode	The nomenclatural code (or codes in the case of an ambiregnal name) under which the scientificName is constructed Recommended best practice is to use a controlled vocabulary.
taxonomicStatus	The status of the use of the scientificName as a label for a taxon Requires taxonomic opinion to define the scope of a taxon Rules of priority then are used to define the taxonomic status of the nomenclature contained in that scope, combined with the experts opinion It must be linked to a specific taxonomic reference that defines the concept Recommended best practice is to use a controlled vocabulary.

## Supplementary Material

Supplementary material 1 Occurence of Irregular Echinoids in mexican waters Data type: occurences, taxonomic
Brief description: The dataset provides the occurence of Irregular Echinoids species in mexican waters, reported in Darwin Core format and published in OBIS http://www.iobis.org/. This file includes the reports from 4 biologic collections (1.ICML-UNAM, 2.USNM, 3.MCZ, 4.YPM).
File: oo_92782.xlsx Martínez-Melo, Solís-Marín, Buitrón-Sánchez & Laguarda-Figueras

## Figures and Tables

**Figure 2.**
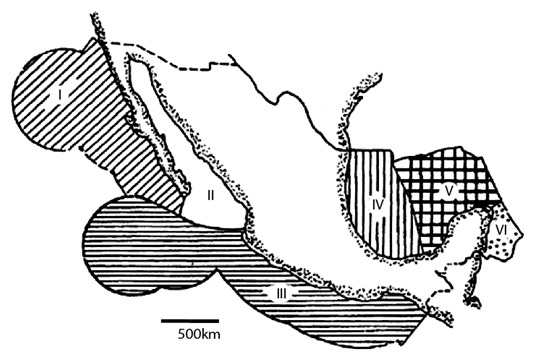
Marine Provinces where the species were reported: I. Lower California-Pacífic, II. Gulf of California, III. Panamic Region of the Pacific Ocean, IV. Southwest of the Gulf of Mexico, V. Bank of Campeche, VI. Mexican Caribbean (taken from Aguayo and Trápaga 1996).

**Table 1.** Species of Irregular Echinods (Echinodermata: Irregularia) from Mexico. Collections where the species were located: 1. ICML-UNAM, 2. USNM, 3. MCZ, 4. YPM. Marine Provinces where the species were reported: I. Lower California-Pacífic, II. Gulf of California, III. Panamic Region of the Pacific Ocean, IV. Southwest of the Gulf of Mexico, V. Bank of Campeche, VI. Mexican Caribbean. See Fig. 2.

**ORDER**	**FAMILY**	**GENUS**	**SPECIES**	**1**	**2**	**3**	**4**	**I**	**II**	**III**	**IV**	**V**	**VI**
**Echinoneoida H. L. Clark, 1925**													
	Echinoneidae L. Agassiz & Desor, 1847												
		*Echinoneus* Leske, 1778											
			*Echinoneus cyclostomus* Leske, 1778	*	*	*						*	*
**Cassiduloida Claus, 1880**													
	Cassidulidae L. Agassiz & Desor, 1847												
		*Cassidulus* Lamarck, 1801											
			*Cassidulus caribaearum* Lamarck, 1801; 1218	*									*
		*Rhyncholampas* A. Agassiz, 1869											
			*Rhyncholampas pacificus* (A. Agassiz, 1863)	*	*	*	*		*	*			
**Echinolampadoida Kroh & Smith, 2010**													
	Echinolampadidae Gray, 1851												
		*Conolampas* A. Agassiz, 1883											
			*Conolampas sigsbei* (A. Agassiz, 1878)	*	*	*						*	*
		*Echinolampas* Gray, 1825											
			*Echinolampas depressa* Gray, 1851	*	*	*						*	*
**Clypeasteroida L. Agassiz, 1835**													
	Clypeasteridae L. Agassiz, 1835												
		*Clypeaster* Lamarck, 1801											
			*Clypeaster chesheri* Serafy, 1970	*								*	*
			*Clypeaster europacificus* H. L. Clark, 1914	*	*	*			*				
			*Clypeaster ochrus* H. L. Clark, 1914	*	*	*			*	*			
			*Clypeaster prostratus* (Ravenel, 1845)	*					*				
			*Clypeaster ravenelii* (A. Agassiz, 1869)	*	*	*					*	*	*
			Clypeaster rosaceus (Linnaeus, 1758)	*	*							*	*
			*Clypeaster rotundus* (A. Agassiz, 1863)	*	*	*			*	*			
			*Clypeaster speciosus* Verrill, 1870	*	*	*	*	*	*				
			*Clypeaster subdepressus* (Gray, 1825)	*	*							*	*
	Fibulariidae Gray, 1855												
		*Echinocyamus* van Phelsum, 1774											
			*Echinocyamus grandiporus* Mortensen, 1907		*							*	*
			*Echinocyamus macrostomus* Mortensen, 1907		*								*
	Dendrasteridae Lambert, 1900												
		*Dendraster* L. Agassiz, 1847											
			*Dendraster excentricus* (Eschscholtz, 1829)	*	*	*		*	*				
			*Dendraster terminalis* (Grant & Hertlein, 1938)			*			*				
			*Dendraster vizcainoensis* Grant & Hertlein, 1938	*	*			*					
	Mellitidae Stephanini, 1912												
		*Encope* L. Agassiz, 1840											
			*Encope aberrans* Martens, 1867	*								*	*
			*Encope grandis* L. Agassiz, 1841	*	*	*	*		*				
			*Encope michelini* L. Agassiz, 1841	*	*	*					*	*	*
			*Encope micropora* L. Agassiz, 1841	*	*	*	*	*	*	*			
			*Encope perspectiva* L. Agassiz, 1841	*	*	*		*	*	*			
			*Encope wetmorei* A. H. Clark, 1946	*	*	*		*	*	*			
		*Leodia* Gray, 1851											
			*Leodia sexiesperforata* (Leske, 1778)	*									*
		*Mellita* L. Agassiz, 1841											
			*Mellita grantii* Mortensen, 1948	*	*				*				
			*Mellita kanakoffi* Durham, 1961	*					*				
			*Mellita longifissa* Michelin, 1858	*	*	*		*	*	*			
			*Mellita notabilis* H. L. Clark, 1947	*	*					*			
			*Mellita quinquiesperforata* (Leske, 1778)	*	*						*		
**Holasteroida Durham & Melville, 1957**													
	Urechinidae Duncan, 1889												
		*Cystechinus* A. Agassiz, 1879											
			*Cystechinus giganteus* A. Agassiz, 1898		*	*			*				
			*Cystechinus loveni* (A. Agassiz, 1898)		*	*		*		*			
		*Urechinus* A. Agassiz, 1879											
			*Urechinus reticulatus* H. L. Clark, 1913		*				*				
	Plaexechinidae Mooi & David, 1996												
		*Plexechinus* A. Agassiz, 1898											
			*Plexechinus cinctus* A. Agassiz, 1898			*			*				
	Pourtalesiidae A. Agassiz, 1881												
		*Cystocrepis* Mortensen, 1907											
			*Cystocrepis setigera* (A. Agassiz, 1898)		*	*				*			
		*Pourtalesia* A. Agassiz, 1869											
			*Pourtalesia tanneri* A. Agassiz, 1898		*	*			*				
**Spatangoida L. Agassiz, 1840**													
	Schizasteridae Lambert, 1905												
		*Aceste* Thomson, 1877											
			*Aceste bellidifera* Thomson, 1877	*							*		
		*Brisaster* Gray, 1855											
			*Brisaster latifrons* (A. Agassiz, 1898)		*	*			*				
			*Brisaster townsendi* (A. Agassiz, 1898)	*	*	*			*				
		*Hypselaster* H. L. Clark, 1917											
			*Hypselaster limicolus* (A. Agassiz, 1878)	*	*						*	*	
		*Moira* A. Agassiz, 1872											
			*Moira atropos* (Lamarck, 1816)	*							*	*	
			*Moira clotho* (Michelin, 1855)	*	*	*			*				
		*Schizaster* L. Agassiz, 1836											
			*Schizaster floridiensis* Kier & Grant, 1965	*									*
	Prenasteridae Lambert, 1905												
		*Agassizia* Valenciennes, 1846											
			Agassizia excentrica A. Agassiz, 1869		*								*
			*Agassizia scrobiculata* Valenciennes, 1846	*	*	*		*	*	*			
	Paleopneustidae A. Agassiz, 1904												
		*Paleopneustes* A. Agassiz, 1873											
			*Paleopneustes tholoformis* Chesher, 1968		*								*
	Palaeotropidae Lambert, 1896												
		*Palaeobrissus* A. Agassiz, 1883											
			*Palaeobrissus hilgardi* A. Agassiz, 1883		*								*
	Brissidae Gray, 1855												
		*Brissopsis* L. Agassiz, 1840											
			*Brissopsis alta* Mortensen, 1907	*	*						*		
			*Brissopsis atlantica* Mortensen, 1907	*	*						*	*	*
			*Brissopsis columbaris* A. Agassiz, 1898	*	*				*				
			*Brissopsis pacifica* (A. Agassiz, 1898)	*	*	*		*	*				
		*Brissus* Gray, 1825											
			*Brissus latecarinatus* (Leske, 1778)			*			*				
			*Brissus obesus* Verrill, 1867	*	*	*	*		*	*			
			*Brissus unicolor* (Leske, 1778)	*	*						*	*	*
		*Meoma* Gray, 1851											
			Meoma ventricosagrandis Gray, 1851	*	*	*			*	*			
			*Meoma ventricosa ventricosa* (Lamarck, 1816)	*	*						*	*	*
		*Metalia* Gray, 1851											
			*Metalia nobilis* Verrill, 1867	*					*				
			*Metalia spatagus* (Linnaeus, 1758)		*	*			*				
		*Neopneustes* Duncan, 1889											
			*Neopneustes micrasteroides* (Duncan, 1889)		*								*
		*Plagiobrissus* Pomel, 1883											
			*Plagiobrissus grandis* (Gmelin, 1788)	*	*							*	*
			*Plagiobrissus pacificus* H. L. Clark, 1940	*					*				
		*Rhynobrissus* Agassiz, 1872											
			*Rhynobrissus cuneus* Cooke, 1957	*						*			
	Spatangidae Gray, 1825												
		*Plethotaenia* H. L. Clark, 1917											
			*Plethotaenia angularis* Chesher, 1968		*								*
			*Plethotaenia spatangoides* (A. Agassiz, 1883)	*	*						*	*	*
		*Spatangus* H. L. Clark, 1917											
			*Spatangus californicus* H. L. Clark, 1917	*	*								*
	Loveniidae Lambert, 1905												
		*Homolampas* Agassiz, 1874											
			*Homolampas fragilis* (A. Agassiz, 1869)		*								*
		*Lovenia* Desor, 1847											
			*Lovenia cordiformis* A. Agass										
